# Transverse Myelitis in a Healthy Adult Female Patient: A Rare Viral Etiology

**DOI:** 10.7759/cureus.73932

**Published:** 2024-11-18

**Authors:** Amina Namrouti, Mary DesRosiers, Claudette Barreto, Ian Brandon, Tate B Hodges, Tariq Mahmood

**Affiliations:** 1 Herbert Wertheim College of Medicine, Florida International University, Miami, USA; 2 Family Medicine, Baptist Health South Florida, Miami, USA; 3 Neuroradiology, Radiology Associates of South Florida, Miami, USA; 4 Neuroradiology, Baptist Health South Florida, Miami, USA; 5 Internal Medicine, Baptist Health South Florida, Miami, USA

**Keywords:** acute cervical transverse myelitis, covid-19, coxsackie b virus, neuroimmunology, spinal cord inflammation

## Abstract

Transverse myelitis (TM) is an uncommon but extensively documented neurological disorder marked by acute spinal cord inflammation, resulting in a spectrum of impairments including motor, sensory, and autonomic disturbances. We outline a rare case of viral-induced TM linked to COVID-19 and Coxsackie B viral infections. A 25-year-old healthy female patient presented with acute bilateral upper and lower extremity weakness, paresthesia, and urinary retention. MRI of the cervical spine revealed abnormal marrow signal intensity. Laboratory investigation highlighted the presence of SARS-CoV-2 RNA; Coxsackie B virus subtypes 1, 2, 4, 5, and 6; and positive Lyme disease IgG subtype. Lumbar puncture and systemic autoimmune cases of myelopathy were unrevealing. Treatment included high-dose steroids, plasmapheresis, Foley catheter placement, and continual physical therapy. Despite improvements, the patient did not reach baseline and was discharged for outpatient physical/occupational rehabilitation. To our knowledge, this is the only case that describes viral-induced TM associated with COVID-19, Coxsackie B, and Lyme disease simultaneously. The rarity of this case highlights the necessity for further investigation into the pathogenesis of viral-induced TM and whether viral causes contribute synergistically or independently to the onset of TM.

## Introduction

Transverse myelitis (TM) is a rare neurological condition characterized by acute spinal cord inflammation that results in damage to the myelin sheath overlying the spinal cord and nerve fibers [1]. This disrupts the ascending and descending nerve pathways, consequently leading to neurological deficits, which include motor, sensory, and autonomic disturbances often presenting as bowel/bladder dysfunction [1].

Diagnosis requires the exclusion of a compressive cord lesion through brain and spinal imaging, followed by confirmation of inflammation through cerebral spinal fluid (CSF) analysis, where pleocytosis or elevated immunoglobulin G (IgG) index would be expected. In addition, gadolinium-enhanced MRI is utilized to exclude a compressive cord lesion [1,2].

Treatment involves the prompt initiation of high-dose intravenous glucocorticoids; plasma exchange may also be beneficial in some cases [3]. Rehabilitation, including physical and occupational therapy, is crucial for maximizing recovery and aims to address motor deficits, enhance mobility, and optimize the quality of life for affected individuals [3].

Although the etiology behind TM remains unclear, various infections and immunologic triggers have been documented and are associated with TM onset. These include parainfectious triggers, autoimmune diseases such as multiple sclerosis (MS), neuromyelitis optica spectrum disorder (NMOSD), acute demyelinating encephalomyelitis (ADEM), and systemic lupus erythematosus (SLE) [1,2].

Limited literature exists on the concurrent association of COVID-19 and Coxsackie B viruses with TM onset and outcomes. We present a rare and significant case of viral-induced TM associated with simultaneous COVID-19 and Coxsackie B infections.

## Case presentation

A previously healthy, right-handed female patient in her mid-20s presented to the emergency department with acute-onset paresthesia, weakness of bilateral upper and lower extremities (L>R), and urinary retention. The onset of symptoms followed a sharp stabbing sensation from the cervical spine that radiated to the lumbar region but only lasted a few seconds. Pain in the cervical neck persisted and was unresponsive to nonsteroidal anti-inflammatory drugs. It was associated with progressive worsening of motor weakness and paresthesia, prompting a visit to the emergency department. No trauma or injury preceded the symptoms, and the patient denied any significant family or social history.

Upon initial evaluation in the emergency department, the patient was afebrile, normotensive, and tachycardic (104 beats per minute), with a respiratory rate of 18 breaths per minute and an SpO2 of 97%-100% on room air. The patient had reported flu-like symptoms, including malaise, sore throat, and congestion, which began the night before, as well as three episodes of non-bloody emesis. She denied blurry/double vision, shortness of breath, or dysphagia. Notably, multiple family members tested positive for COVID-19, and a respiratory panel identified SARS-CoV-2 RNA. Chest X-ray showed no acute chest abnormalities. A decision to defer remdesivir administration was made due to the absence of respiratory symptoms. The patient was found to have 800 cc of retained urine on a bladder scan, which prompted the placement of a Foley catheter. She denied any prior episodes of urinary incontinence or retention.

Initial neurological examination revealed diminished sensation at and below the T4 dermatome level, with bilateral shoulder strength 4/5, wrist weakness and wrist drop noted (L>R: left wrist extension/flexion 1/5, right wrist extension/flexion 2/5), and bilateral hips/knees 3/5. Reflexes were 2+/5 in the bilateral upper extremities and 3+/5 in the bilateral lower extremities, with ankle clonus and slow finger-to-nose coordination bilaterally (L>R).

Laboratory investigations revealed leukocytosis of 15,000 white blood cells and elevated C-reactive protein at 16.6 µg/mL (reference 0-10), illustrating the presence of some infectious etiologies. However, other parameters including vitamin B12 and serum complement levels (C1, C2, C3, and C5) were within normal limits. Further testing for HIV I/II, HTLV I/II, EBV, rapid plasma reagin (RPR), group A *Streptococcus*, and acute urinary infections yielded negative results (Table 1 and Table 2). Additional investigation for autoimmune antibodies including antinuclear antibody (ANA), lupus double-stranded DNA, Sjogren’s anti-Ro (SS-A) and anti-La (SS-B), neuromyelitis optica (NMO), and myelin oligodendrocyte glycoprotein (MOG) were nonrevealing (Table 1). Complete viral serology was notably only positive for Coxsackie B type 1 (1:8), type 2 (1:16), type 4 (1:8), type 5 (1:8), and type 6 (1:16), as well as one IgG Lyme disease antibody subtype of the 16 tested (IgG LymeC-P93) (Table 2).

**Table 1:** Serum studies conducted during the evaluation with the patient, including autoimmune antibodies, complement levels, coagulation levels, and reference values WBC: white blood cell, INR: international normalized ratio, PT: prothrombin time, PTT: partial thromboplastin time.

Serum studies	Patient's value	Reference values
Leukocyte count (WBC) on admission	15.25	3.4-11.0 K/µL
Leukocyte count range	13.32-18.96	3.4-11.0 K/µL
C-reactive protein	16.6	0-10 µg/mL
Vitamin B12	682	193-986 pg/mL
Complement C3	116	90-180 mg/dL
Complement C1	14.2	10.3-20.5 mg/dL
Complement C2	2.6	1.4-3.3 mg/dL
Complement C5	15	7-20 mg/dL
Antinuclear antibodies	Negative	≤1:40
Anti-double-stranded DNA antibodies	1 IU/mL	0-9 /mL
Anti-Ro (SS-A)	<0.2 AI	0.0-0.9 AI
Anti-La (SS-B)	<0.2 AI	0.0-0.9 AI
Neuromyelitis optica (NMO) antibodies	<1.5 U/mL	0.0-3.0 U/mL
Myelin oligodendrocyte glycoprotein (MOG) antibodies	<1:1600	<1:1600 titer
Fibrinogen	<60	200 to 400 mg/dL
INR	1.9	1 second
PT	22.7	11-13 seconds
PTT	38.5	21.8-39.0

**Table 2:** Patient's viral serology panel This table illustrates the viral serology panel done during the patient's evaluation, illustrating the pan-positive Coxsackie B viral subtypes, along with the COVID-19 nasopharyngeal swab, and one Lyme IgG serotype.

Viral serology	Patient's value	Reference value
Coxsackie B type 1	1:8	Negative: <1:8
Coxsackie B type 2	1:16	Negative: <1:8
Coxsackie B type 3	Test not performed, unavailability of reagents	Negative: <1:8
Coxsackie B type 4	1:8	Negative: <1:8
Coxsackie B type 5	1:8	Negative: <1:8
Coxsackie B type 6	1:16	Negative: <1:8
LymeC-P93 Ab IgG CSF	Present	Absent
SARS-CoV-2 (COVID-19)	Detected	Not detected

**Figure 1:**
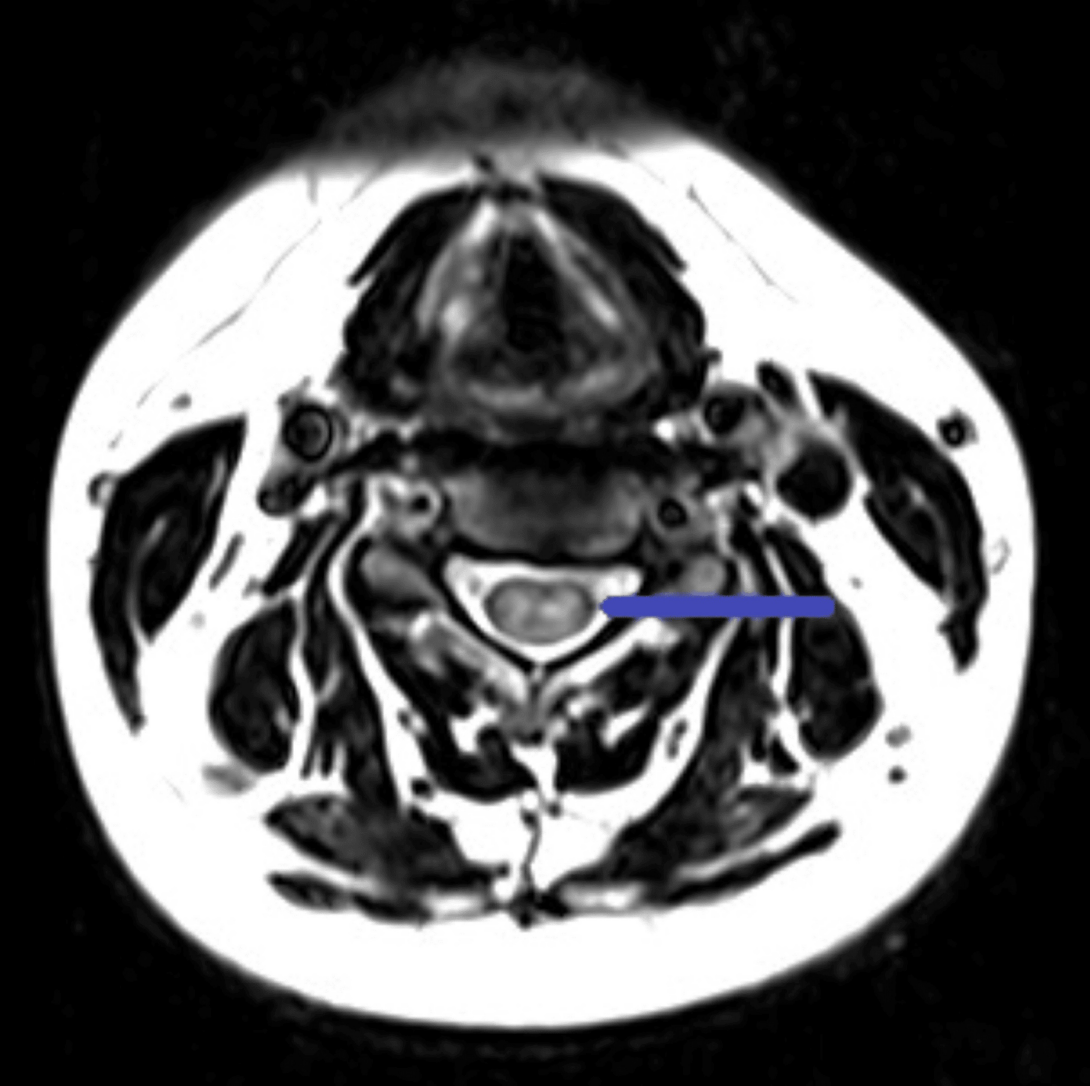
Axial T2-weighted image demonstrating a central pattern of abnormal T2 hyperintense signal (blue arrow) within the spinal cord, occupying greater than two-thirds of the cross-sectional area of the cord. This is a common feature of transverse myelitis.

**Figure 2:**
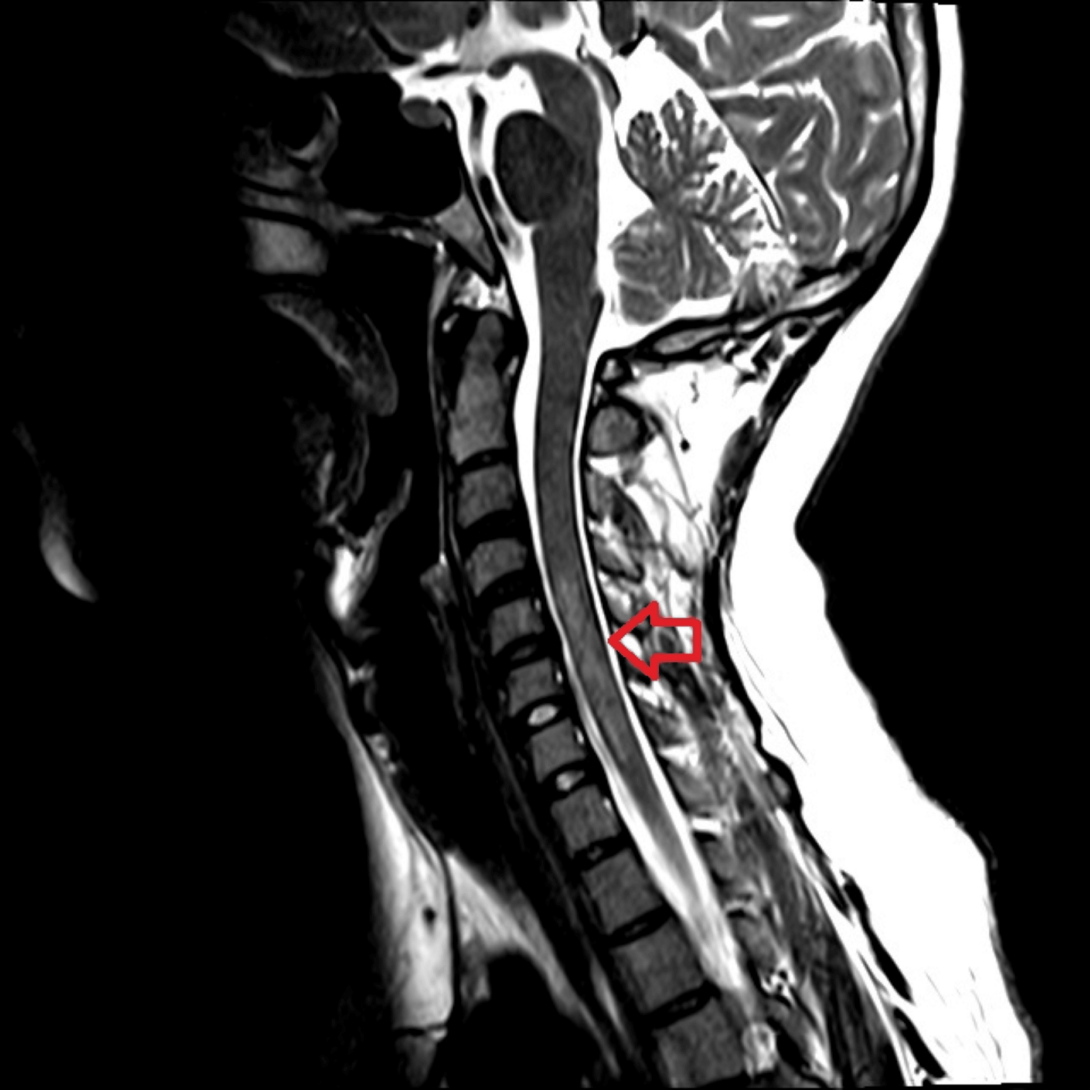
Sagittal T2-weighted image demonstrating a longitudinal band of abnormal hyperintense signal within the spinal cord, spanning from C4-C5 through C7-T1 (red arrow), with minimal associated cord edema/swelling. Involvement of the spinal cord spanning three or more vertebral body segments, as in this case, is typical in transverse myelitis.

**Figure 3:**
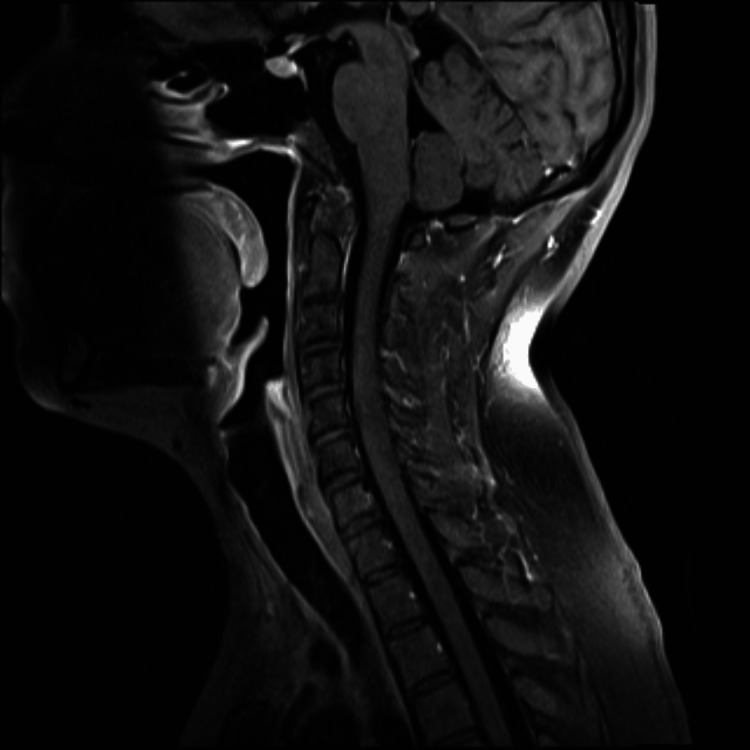
Sagittal T1 postcontrast image demonstrating no abnormal enhancement within the spinal cord. Contrast enhancement is variable in cases of transverse myelitis.

Imaging studies, including CT/MRI of the brain and CT of the cervical spine, were unremarkable. However, cervical spine MRI with/without contrast revealed abnormal marrow signal intensity in the mid to lower cervical cord, suggestive of TM (Figures 1-3). Thoracic spine MRI was unremarkable, except for incidental striated kidney enhancements, warranting renal ultrasound evaluation, which showed no abnormalities. Urinalysis and kidney function on labs were unremarkable, and the patient denied any urinary symptoms. Lumbar puncture was unrevealing: WBCs 2, RBCs 3, lymphocytes 33%, monocytes 67%, total protein 17, and glucose 81, with a negative meningoencephalitis panel (Table 3). In addition, CSF was negative for oligoclonal bands, vitamin E, angiotensin, copper, and cryptococcal antigen, further ruling out autoimmune, infectious, or metabolic pathologies (Table 3).

**Table 3:** Cerebrospinal fluid analysis This table provides the CSF analysis conducted during the patient's evaluation, illustrating pleocytosis with normal glucose and protein levels. In addition, it demonstrates the various other CSF studies conducted during evaluation, including vitamin levels, hard metal, and multiple sclerosis evaluation.

Cerebrospinal fluid	Patient's value	Reference values
Color	No color	No color
Appearance	Clear	Clear
Leukocytes	2	0-5 cells/µL
Erythrocytes	3	0 cells/µL
Lymphocytes	33%	28%-96%
Monocytes	67%	16%-56%
Total protein	17	15-45 mg/dL
Glucose	81	40-75 mg/dL
Vitamin E (alpha-tocopherol)	8.1	5.9-19.4 mg/L
Angiotensin-converting enzyme	<1.5	0.0-2.5 units/L
Copper	107	80-158 µg/dL
Zinc	39	44-115
Cryptococcal antigen	Negative	Negative
Oligoclonal bands	0	Criteria for positivity 4+ oligoclonal bands

The patient was transferred to the intensive care unit with neurological checks bi-hourly. One gram of intravenous methylprednisolone was initiated daily for a total of five days. The patient subsequently demonstrated persistent weakness and new-onset left foot drop. Consequently, plasmapheresis with albumin bi-daily for five days was initiated, which led to significant improvements in lower extremity motor strength (5/5) and shoulder strength (5/5) and resolution of foot drop and clonus. However, left wrist drop and weakness in bilateral upper extremities persisted. 

Following the third course of plasmapheresis, the patient developed coagulopathy, with decreased fibrinogen (<60), elevated international normalized ratio (INR; 1.9), and elevated prothrombin time (PT; 22.7). Hematology was thus consulted, and the coagulopathic state was attributed to consumptive coagulopathy secondary to plasmapheresis. Clotting parameters normalized following the administration of cryoprecipitate transfusion twice (Table 4).

**Table 4:** Coagulation studies prior to and following correction with cryoprecipitate This table illustrates the patient's coagulation panel following plasmapheresis prior to cryoprecipitate administration. Values reveal low fibrinogen with elevated PT and PTT, demonstrating a coagulopathic panel. Values normalized following cryoprecipitate administration. INR: international normalized ratio, PT: prothrombin time, PTT: partial thromboplastin time.

S/P cryoprecipitate	Patient's value	Reference values
Fibrinogen	<60 --> 212	200-400 mg/dL
PT	22.7 --> 14.7	11-13 seconds
INR	1.9 --> 1.1	1 second
PTT	28.1	21.8-39.0

Throughout hospitalization, the patient tolerated treatment and received intensive physical/occupational therapy. Foley catheterization was maintained due to urinary retention, and manual disimpaction was required for fecal management. Persistent leukocytosis was noted throughout her stay, ranging 13,000-18,000, and was attributed secondary to high-dose steroids by hematology. The patient was afebrile throughout her admission, with no new symptomologies.

Despite neurological improvements, the patient did not reach baseline and was discharged for outpatient physical/occupational rehabilitation, with a Foley catheter in place. She was discharged with persistent intrinsic left-hand muscle weakness, bilateral reduced hand grasp L (1/5) > R (2/5), and persistent upper motor neuron signs, including hyperreflexia to bilateral lower extremities.

At three weeks of follow-up, the patient reported complete restoration of strength in the lower extremities and right upper extremity, regained bowel function, and improved bladder function and oxybutynin. Left upper extremity weakness persisted but was progressively improving, and she was placed on baclofen for some residual spasticity. The patient resumed activities of daily living with minimal help and continues to see outpatient physical/occupation therapy twice weekly.

## Discussion

Coxsackie B virus, associated with various neurological conditions, including aseptic meningitis and encephalitis, has growing recognition for its neurotropic potential, but the literature documenting its association with TM onset is limited [3]. Current existing cases document positivity for only one or two Coxsackie B serotypes [4]. Our study identified positivity for five out of the six Coxsackie B serotypes, a finding uncommon in published literature. The absence of the sixth serotype, owing to laboratory material unavailability, presents a limitation. However, given the consistent positivity observed across the other five serotypes, it is reasonable to infer that the sixth would likely have yielded a positive result as well. The elevated levels and diffuse positivity of Coxsackie B virus in our patient strongly suggest its potential role as one of the instigators of TM. It remains unclear how Coxsackie B causes these CNS complications, but proposed mechanisms include viral inhibition of cellular macromolecular synthesis, viral protein toxicity, and apoptosis [3].

A growing number of cases have highlighted the association between COVID-19 and the onset of TM. It is important to note, however, that the majority of the reported cases documented the onset of TM disease with COVID-19 post-vaccination or post-infection, with only a few highlighting the association with active COVID-19 infection as in this patient [5,6]. The precise mechanism of SARS-CoV-2-induced neurological damage remains unclear, but a possible pathway includes direct invasion into the spinal cord facilitated by the presence of angiotensin-converting enzyme 2 (ACE-2) receptors on neurons [7]. Another potential mechanism involves the cytokine storm associated with viral infections, which may result in elevated immunological factors and possibly trigger autoimmune responses [7].

Our patient's positive SARS-CoV-2 RNA nasopharyngeal swab, coupled with symptoms of malaise, sore throat, and a history of sick contacts, suggests an active COVID-19 infection. The simultaneous onset of TM with the manifestation of COVID-19 symptoms suggests the possibility of COVID-19 being one of the potentiators of TM onset in this case.

The positive Lyme IgG antibody raises the possibility of Lyme disease as a potential trigger. However, it is important to note that the IgG antibody may indicate past exposure rather than acute infection. Additionally, only one antibody out of the 16 tested was positive. Given the absence of other clinical features suggestive of Lyme disease and the unrevealing cerebrospinal fluid analysis, the contribution of Lyme disease to the development of TM in this case appears less likely.

Given the absence of positive autoimmune panels and medical history, coupled with the patient's active COVID-19 infection and a pan-positive Coxsackie B panel, the most likely etiology for the onset of TM appears to be the concurrent viral infections. 

The simultaneous detection of these agents complicates the determination of their individual roles in disease onset/progression, as they may have acted synergistically or independently; thus, it remains unclear which of them, if any, exerted a more substantial influence on the induction/onset of the inflammatory process associated with TM.

## Conclusions

To our knowledge, this is the first documented case in which multiple infectious agents, such as Coxsackie B viruses, SARS-CoV-2, and Lyme disease, were detected concurrently, highlighting the intricate nature of TM causation. This case emphasizes the importance of recognizing TM in patients with COVID-19 and Coxsackie B infections, to ensure close neurological monitoring and the prompt initiation of appropriate treatment to prevent disease complications. The rarity of this case calls for further investigation into the pathogenesis of viral-induced TM and whether these viral agents contribute synergistically or independently to the onset of TM.

## Appendices

Supplemental material 

**Table 5:** Cerebrospinal fluid meningoencephalitis panel CSF PCR: cerebrospinal fluid polymerase chain reaction.

Meningoencephalitis panel	Patient's value	Reference value
*Escherichia coli* by K1 CSF PCR	Not detected	Not detected
*Haemophilus influenzae* CSF PCR	Not detected	Not detected
*Listeria monocytogenes* CSF PCR	Not detected	Not detected
*Neisseria meningitidis* CSF PCR	Not detected	Not detected
*Streptococcus agalactiae* CSF PCR	Not detected	Not detected
*Streptococcus pneumoniae* CSF PCR	Not detected	Not detected
*Cytomegalovirus *CSF PCR	Not detected	Not detected
*Enterovirus *CSF PCR	Not detected	Not detected
Herpes simplex virus 1 CSF PCR	Not detected	Not detected
Herpes simplex virus 2 CSF PCR	Not detected	Not detected
Human herpesvirus 6 (HHV-6) CSF PCR	Not detected	Not detected
Human *Parechovirus* CSF PCR	Not detected	Not detected
Varicella zoster virus CSF PCR	Not detected	Not detected
*Cryptococcus neoformans/gattii* CSF PCR	Not detected	Not detected

**Table 6:** Infectious disease workup HIV: human immunodeficiency virus, HTLV: human T-lymphotropic virus, RPR: rapid plasma reagin, VDRL-CSF: venereal disease research laboratory test of the cerebrospinal fluid.

	Patient's value	Reference value
HIV I/II	Nonreactive	Nonreactive
HTLV I/II	Negative	Negative
RPR	Nonreactive	Nonreactive
VDRL-CSF	Nonreactive	Nonreactive
Monospot test	Negative	Negative
